# Supplementary material for: Functional and evolutionary analysis of host Synaptogyrin-2 in porcine circovirus type 2 susceptibility
Source: PLoS Genet. 2023 Nov 27;19(11):e1011029. doi: 10.1371/journal.pgen.1011029 (PMC10703400; doi:10.1371/journal.pgen.1011029)
Supplement: S1 Text — Fig A. CRISPR-Cas9 guide RNA and template design for generation of clones homozygous for alternate SYNGR2 p.63Cys allele. The selected guide RNA (sg_AF) targeted a PAM site within exon 2 (gray box) that included the SYNGR2 p.63Arg allele (red). An 80 bp ssDNA sequence (thick black line) homologous to the non-targeting strand and encoding the alternate SYNGR2 p.63Cys allele (yellow) was used as a template for homology-directed repair. Fig B. Alignment of the SYNGR2 coding sequence for wildtype and edited PK15 cell lines. Sequences were obtained by targeted amplification of the SYNGR2 transcript and Sanger sequencing. Position of the substitution of p.63Arg (C) to p.63Cys (T) in the edited PK15 clones, emSYNGR2+p.63Cys and 2emSYNGR2+p.63Cys, is highlighted in red. The 106 bp deletion in the predicted SYNGR2 knock-out PK15 clone, emSYNGR2-del, is represented by dashes. Fig C. Expression of SYNGR2 in wildtype and edited PK15 following PCV2b infection mock-infected control cells. Expression represented as Log10 transformed mean normalized expression (MNE) across three independent replicates with error bars representing one standard error from the mean. Samples collected from control and infected cells across timepoints post PCV2b infection (MOI = 0.00075). Letters denote significant differences in gene expression between cell lines within treatment group (C = control, I = infected) or between treatment groups within cell lines (wt = wildtype, em = edited). *P<0.05, **P<0.01. Fig D. Frequency of SYNGR2 haplotypes across geographic and domestic/wild S. scrofa subgroups. The two haplotypes that differ by only the SYNGR2 p.Arg63Cys allele, Hap1 (Cys) and Hap2 (Arg), are represented as independent pie segments. Hap3-Hap10 and rare haplotypes were combined into a single category denoted as “Other”. (ASW = Asian Wild Boar, ASD = Asian Domestic, EUW = European Wild Boar, EUD = European Domestic). Fig E. Frequency of SYNGR2 haplotypes across domestic breeds. The two haplotypes th [file pgen.1011029.s001.docx]

**Fig A.** **CRISPR-Cas9 guide RNA and template design for generation of clones homozygous for alternate *SYNGR2 p.63Cys* allele.** The selected guide RNA (sg_AF) targeted a PAM site within exon 2 (gray box) that included the *SYNGR2 p.63Arg* allele (red). An 80 bp ssDNA sequence (thick black line) homologous to the non-targeting strand and encoding the alternate *SYNGR2 p.63Cys* allele (yellow) was used as a template for homology-directed repair.


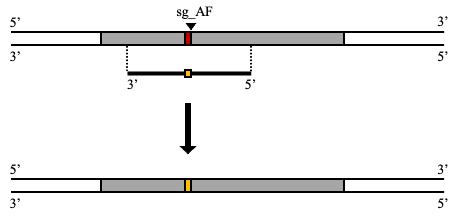


**Fig B. Alignment of the *SYNGR2* coding sequence for wildtype and edited PK15 cell lines.** Sequences were obtained by targeted amplification of the *SYNGR2* transcript and Sanger sequencing. Position of the substitution of *p.63Arg* (C) to *p.63Cys* (T) in the edited PK15 clones, *emSYNGR2^+p.63Cys^* and *2emSYNGR2^+p.63Cys^*, is highlighted in red. The 106 bp deletion in the predicted *SYNGR2* knock-out PK15 clone, *emSYNGR2^-del^*, is represented by dashes.


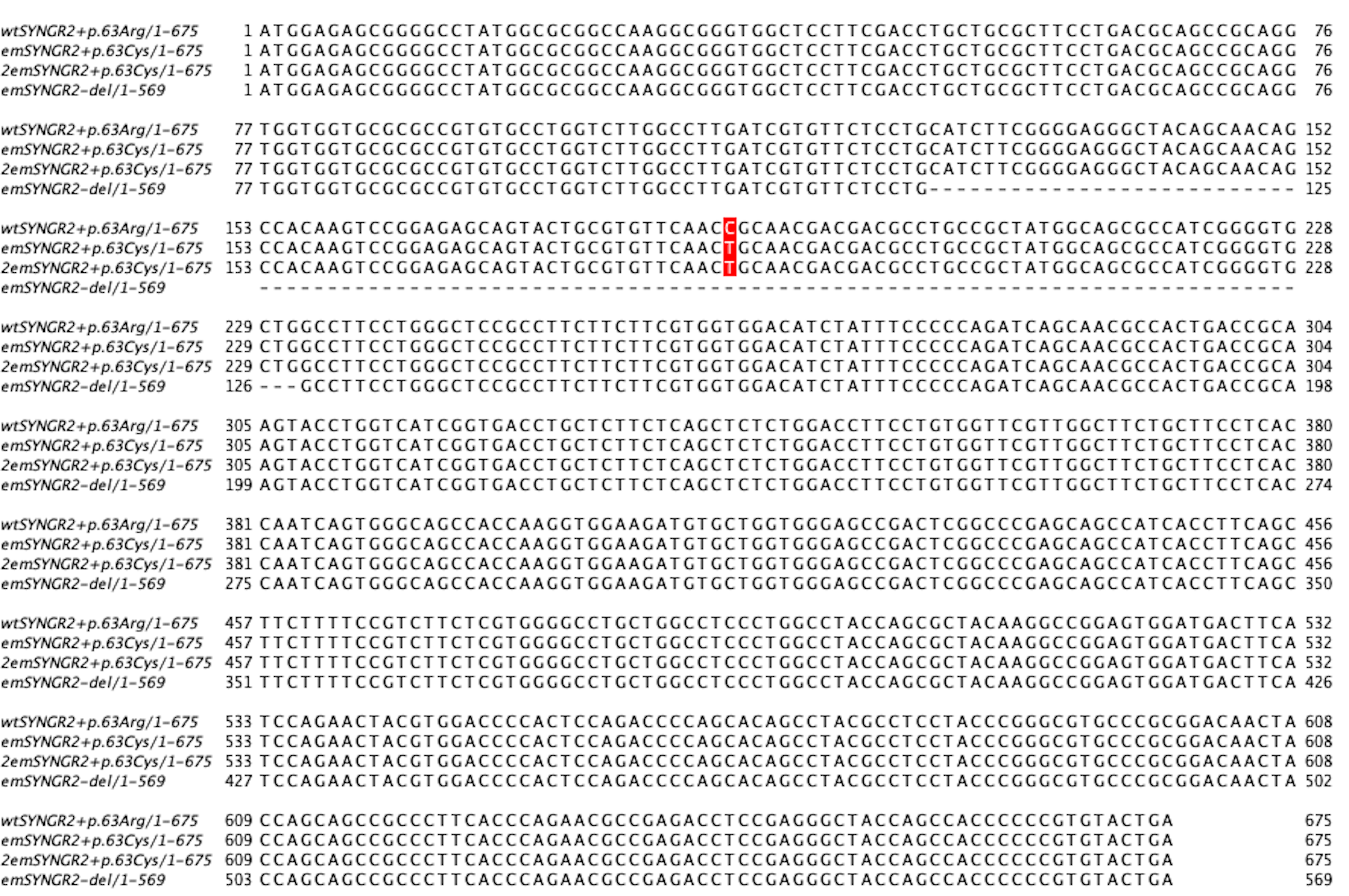


**Fig C. Expression of *SYNGR2* in wildtype and edited PK15 following PCV2b infection mock-infected control cells.** Expression represented as Log10 transformed mean normalized expression (MNE) across three independent replicates with error bars representing one standard error from the mean. Samples collected from control and infected cells across timepoints post PCV2b infection (MOI=0.00075). Letters denote significant differences in gene expression between cell lines within treatment group (C = control, I = infected) or between treatment groups within cell lines (wt = wildtype, em = edited). *P<0.05, **P<0.01


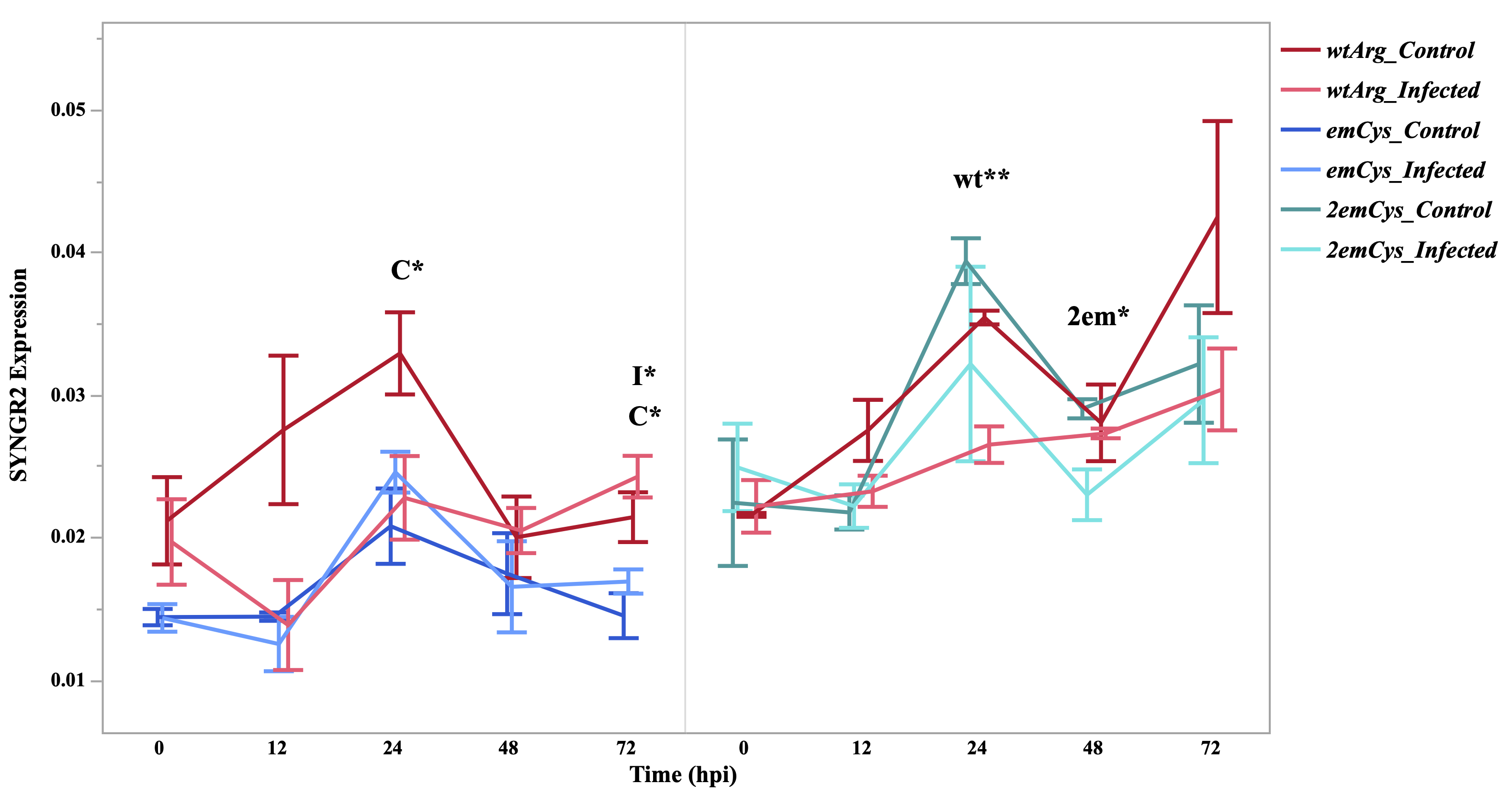


**Fig D. Frequency of *SYNGR2* haplotypes across geographic and domestic/wild *S. scrofa* subgroups.** The two haplotypes that differ by only the *SYNGR2 p.Arg63Cys* allele, *Hap1* (*Cys*) and *Hap2* (*Arg*), are represented as independent pie segments. Hap3-Hap10 and rare haplotypes were combined into a single category denoted as “Other”. (ASW = Asian Wild Boar, ASD = Asian Domestic, EUW = European Wild Boar, EUD = European Domestic)


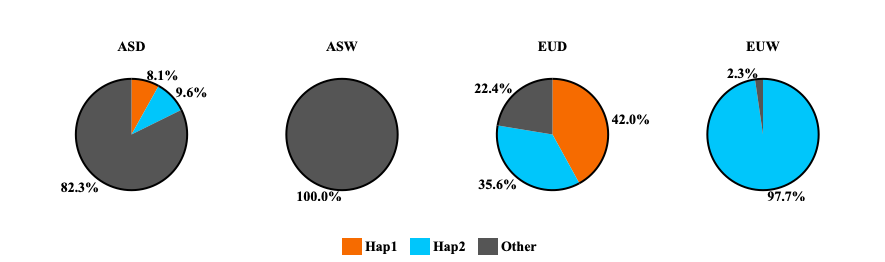


**Fig E.** **Frequency of *SYNGR2* haplotypes across domestic breeds.** The two haplotypes that differ by only the *SYNGR2 p.Arg63Cys* SNP, *Hap1* (*Cys*) and *Hap2* (*Arg*), are represented as independent pie segments. Hap3-Hap10 and rare haplotypes were combined into a single category denoted as “Other”. (BR = Berkshire, DR = Duroc, IB = Iberian, PI = Pietrain, LR = Landrace, LW = Large White, YR = Yorkshire, EUDO = European Domestic Other, EH = Erhualian, MS = Meishan, ASDO = Asian Domestic Other)


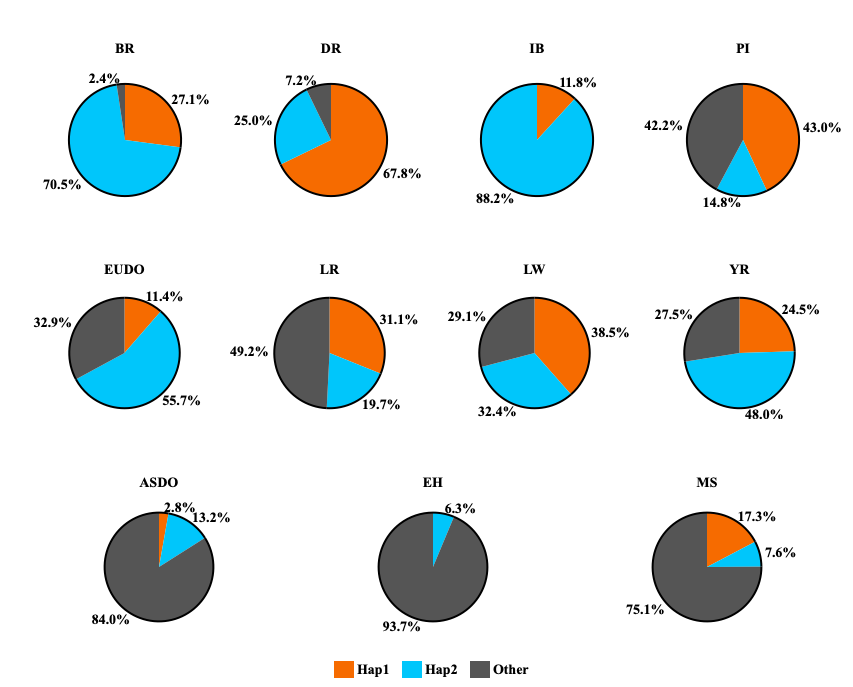


**Table A. Mammalian *SYNGR2* transcript sequences and taxonomic groups**

| **Transcript ID** | **Species** | **Mammals** | **Even-Toed Ungulates** | **Primates** |
| --- | --- | --- | --- | --- |
| XM_012453596.2 | *Aotus nancymaae* |  |  | X |
| NM_001100358.1 | *Bos taurus* | X | X |  |
| XM_025279604.1 | *Bubalus bubalis* |  | X |  |
| XM_002748794.3 | *Calithrix jacchus* |  |  | X |
| XM_010950707.1 | *Camelus bactrianus* |  | X |  |
| XM_025468150.1 | *Canis lupus dingo* | X |  |  |
| XM_018063687.1 | *Capra hircus* | X | X |  |
| XM_020158831.1 | *Castor canadensis* | X |  |  |
| XM_017525004.1 | *Cebus capucinus* |  |  | X |
| XM_012042271.1 | *Cercocebus atys* |  |  | X |
| XM_008013077.2 | *Chlorocebus sabaeus* |  |  | X |
| XM_004460431.3 | *Dasypus novemcinctus* | X |  |  |
| XM_022589471.1 | *Delphinapterus leucas* |  | X |  |
| XM_023651917.1 | *Equus caballus* | X |  |  |
| XM_023244126.1 | *Felis catus* | X |  |  |
| XM_031011362.1 | *Gorilla gorilla gorilla* |  |  | X |
| XM_004860817.2 | *Heterocephalus glaber* | X |  |  |
| NM_004710.7 | *Homo sapien* | X |  | X |
| NM_001266199.1 | *Macaca mulatta* | X |  | X |
| XM_011720052.1 | *Macaca nemestrina* |  |  | X |
| XM_005069858.3 | *Mesocricetus auratus* | X |  |  |
| XM_012756241.2 | *Microcebus murinus* |  |  | X |
| NM_009304.2 | *Mus musculus* | X |  |  |
| XM_004394273.1 | *Odobenus rosmarus divergens* | X |  |  |
| XM_020889014.1 | *Odocoileus virginianus taxanus* |  | X |  |
| XM_004275529.2 | *Orcinus orca* | X | X |  |
| XM_017340214.1 | *Oryctolagus cuniculus* | X |  |  |
| XM_027974188.1 | *Ovis aries* | X | X |  |
| XM_016933021.2 | *Pan troglodytes* | X |  | X |
| XM_019466061.1 | *Panthera pardus* | X |  |  |
| XM_003913513.3 | *Papio anubis* |  |  | X |
| XM_007099806.2 | *Physeter Catodon* |  | X |  |
| XM_023211671.2 | *Piliocolobus tephrosceles* |  |  | X |
| XM_009252079.2 | *Pongo abelii* |  |  | X |
| XM_012661327.1 | *Propithecus coquereli* |  |  | X |
| XM_021066554.1 | *Sus scrofa* | X | X |  |
| XM_025363542.1 | *Theropithecus gelada* |  |  | X |
| XM_004374112.2 | *Trichechus manatus latirostris* | X |  |  |
| XM_026484021.1 | *Ursus arctos horribilis* | X |  |  |
| XM_027850218.1 | *Vombatus ursinus* | X |  |  |

**Table B. *SYNGR2* SNP identified across *Suidae* sequences.** Each *SYNGR2* SNP is denoted by nucleotide position within the *SYNGR2* coding sequence. (*187= *SYNGR2 p.Arg63Cys*; SSC12=chromosome 12, *S.scrofa*11.1)

| ***SYNGR2* SNP** | **Position** | | **Alleles** | | **Predicted Consequence** | **Predicted aa** |
| --- | --- | --- | --- | --- | --- | --- |
|  | SSC12 (bp) | Protein (aa) | Ref | Alt |  |  |
| 18 | 3,799,562 | 6 | T | C | Synonymous | Tyr |
| 105 | 3,797,597 | 35 | G | A | Synonymous | Leu |
| 187* | 3,797,515 | 63 | T | C | Missense | Cys (T) Arg (C) |
| 192 | 3,797,510 | 64 | C | T | Synonymous | Asn |
| 195 | 3,797,507 | 65 | C | T | Synonymous | Asp |
| 198 | 3,797,504 | 66 | C | T | Synonymous | Asp |
| 210 | 3,797,492 | 70 | T | C | Synonymous | Tyr |
| 261 | 3,797,441 | 87 | G | C/T | Synonymous | Val |
| 321 | 3,797,381 | 107 | T | C | Synonymous | Gly |
| 324 | 3,797,378 | 108 | C | T | Synonymous | Asp |
| 465 | 3,796,891 | 155 | C | T | Synonymous | Ser |
| 516 | 3,796,754 | 172 | C | T | Synonymous | Ala |
| 540 | 3,796,733 | 180 | C | T | Synonymous | Asn |
| 564 | 3,796,709 | 188 | C | T | Synonymous | Pro |
| 576 | 3,796,694 | 192 | C | T | Synonymous | Tyr |
| 588 | 3,796,682 | 196 | G | A | Synonymous | Pro |
| 597 | 3,796,673 | 199 | C | T | Synonymous | Pro |
| 603 | 3,796,667 | 201 | C | T | Synonymous | Asp |
| 645 | 3,796,625 | 215 | C | T | Synonymous | Ser |

**Table C. Allelic frequencies for *SYNGR2* SNP across *Sus scrofa* subgroups.** (*187=*SYNGR2 p.Arg63Cys***;** EUD=European Domestic, EUW=European Wild, ASD=Asian Domestic, ASW=Asian Wild)

| **SNP** | **Allele** | **EUD** | **ASD** | **EUW** | **ASW** | **Overall** |
| --- | --- | --- | --- | --- | --- | --- |
| 187* | C (*Arg*) | 0.569 | 0.919 | 1 | 1 | 0.661 |
|  | T (*Cys*) | 0.431 | 0.081 | 0 | 0 | 0.339 |
| 195 | C | 0.999 | 0.938 | 1 | 0.761 | 0.98 |
|  | T | 0.001 | 0.062 | 0 | 0.239 | 0.02 |
| 198 | C | 0.83 | 0.269 | 0.977 | 0.217 | 0.712 |
|  | T | 0.17 | 0.731 | 0.023 | 0.783 | 0.288 |
| 210 | C | 0.024 | 0.004 | 0 | 0.043 | 0.02 |
|  | T | 0.976 | 0.996 | 1 | 0.957 | 0.98 |
| 261 | C | 0.172 | 0.558 | 0.023 | 0.543 | 0.25 |
|  | G | 0.819 | 0.338 | 0.977 | 0.043 | 0.711 |
|  | T | 0.009 | 0.104 | 0 | 0.413 | 0.039 |
| 321 | C | 0.189 | 0.81 | 0.023 | 1 | 0.323 |
|  | T | 0.811 | 0.19 | 0.977 | 0 | 0.677 |
| 465 | C | 0.98 | 0.833 | 1 | 0.75 | 0.948 |
|  | T | 0.02 | 0.167 | 0 | 0.25 | 0.052 |
| 516 | C | 0.983 | 0.762 | 1 | 0.804 | 0.938 |
|  | T | 0.017 | 0.238 | 0 | 0.196 | 0.062 |
| 540 | C | 0.847 | 0.457 | 0.976 | 0.565 | 0.77 |
|  | T | 0.153 | 0.543 | 0.024 | 0.435 | 0.23 |
| 597 | C | 0.864 | 0.682 | 0.977 | 0.727 | 0.83 |
|  | T | 0.136 | 0.318 | 0.023 | 0.273 | 0.17 |
| 603 | C | 0.866 | 0.688 | 0.977 | 0.762 | 0.834 |
|  | T | 0.134 | 0.312 | 0.023 | 0.238 | 0.166 |

| **SNP** | **Allele** | **DR** | **LR** | **LW** | **YR** | **PI** | **IB** | **BR** | **EUDO** | **MS** | **EH** | **ASDO** | **Overall** |
| --- | --- | --- | --- | --- | --- | --- | --- | --- | --- | --- | --- | --- | --- |
| *187 | C (*Arg*) | 0.317 | 0.647 | 0.605 | 0.755 | 0.588 | 0.882 | 0.705 | 0.864 | 0.83 | 1 | 0.972 | 0.638 |
|  | T (*Cys*) | 0.683 | 0.353 | 0.395 | 0.245 | 0.412 | 0.118 | 0.295 | 0.136 | 0.17 | 0 | 0.028 | 0.362 |
| 195 | C | 0.997 | 1 | 1 | 1 | 1 | 1 | 1 | 1 | 0.868 | 1 | 0.981 | 0.987 |
|  | T | 0.003 | 0 | 0 | 0 | 0 | 0 | 0 | 0 | 0.132 | 0 | 0.019 | 0.013 |
| 198 | C | 0.936 | 0.699 | 0.767 | 0.774 | 0.618 | 1 | 1 | 0.716 | 0.387 | 0.125 | 0.217 | 0.721 |
|  | T | 0.064 | 0.301 | 0.233 | 0.226 | 0.382 | 0 | 0 | 0.284 | 0.613 | 0.875 | 0.783 | 0.279 |
| 210 | C | 0.003 | 0.108 | 0.029 | 0 | 0 | 0 | 0 | 0 | 0 | 0 | 0.009 | 0.02 |
|  | T | 0.997 | 0.892 | 0.971 | 1 | 1 | 1 | 1 | 1 | 1 | 1 | 0.991 | 0.98 |
| 261 | C | 0.064 | 0.36 | 0.203 | 0.198 | 0.361 | 0 | 0 | 0.273 | 0.472 | 0.833 | 0.519 | 0.247 |
|  | G | 0.933 | 0.591 | 0.797 | 0.802 | 0.639 | 1 | 1 | 0.727 | 0.387 | 0.167 | 0.368 | 0.725 |
|  | T | 0.003 | 0.048 | 0 | 0 | 0 | 0 | 0 | 0 | 0.142 | 0 | 0.113 | 0.028 |
| 321 | C | 0.073 | 0.414 | 0.222 | 0.189 | 0.389 | 0 | 0.024 | 0.284 | 0.74 | 0.917 | 0.83 | 0.309 |
|  | T | 0.927 | 0.586 | 0.778 | 0.811 | 0.611 | 1 | 0.976 | 0.716 | 0.26 | 0.083 | 0.17 | 0.691 |
| 465 | C | 0.944 | 1 | 1 | 1 | 1 | 1 | 1 | 1 | 0.96 | 0.542 | 0.846 | 0.952 |
|  | T | 0.056 | 0 | 0 | 0 | 0 | 0 | 0 | 0 | 0.04 | 0.458 | 0.154 | 0.048 |
| 516 | C | 0.953 | 1 | 1 | 1 | 1 | 1 | 1 | 0.989 | 0.941 | 0.438 | 0.735 | 0.941 |
|  | T | 0.047 | 0 | 0 | 0 | 0 | 0 | 0 | 0.011 | 0.059 | 0.562 | 0.265 | 0.059 |
| 540 | C | 0.938 | 0.753 | 0.824 | 0.774 | 0.639 | 1 | 1 | 0.682 | 0.548 | 0.146 | 0.509 | 0.771 |
|  | T | 0.062 | 0.247 | 0.176 | 0.226 | 0.361 | 0 | 0 | 0.318 | 0.452 | 0.854 | 0.491 | 0.229 |
| 597 | C | 0.992 | 0.736 | 0.818 | 0.783 | 0.594 | 1 | 1 | 0.705 | 0.615 | 0.688 | 0.745 | 0.829 |
|  | T | 0.008 | 0.264 | 0.182 | 0.217 | 0.406 | 0 | 0 | 0.295 | 0.385 | 0.312 | 0.255 | 0.171 |
| 603 | C | 0.992 | 0.734 | 0.83 | 0.792 | 0.588 | 1 | 1 | 0.705 | 0.608 | 0.688 | 0.764 | 0.831 |
|  | T | 0.008 | 0.266 | 0.17 | 0.208 | 0.412 | 0 | 0 | 0.295 | 0.392 | 0.312 | 0.236 | 0.169 |

**Table D. Allelic frequencies for *SYNGR2* SNP across domestic breeds.** (*187 = *SYNGR2 p.Arg63Cys*; BR=Berkshire, DR=Duroc, IB=Iberian, PI=Pietrain, LR=Landrace, LW=LargeWhite, YR=Yorkshire, EUDO=European other, EH=Erhualian, MS=Meishan, ASDO= Asian other)

**Table E. Frequency of *SYNGR2* haplotypes across *S. scrofa* subgroups. (**EUD=European Domestic, EUW=European Wild, ASD=Asian Domestic, ASW=Asian Wild)

| ***SYNGR2* Haplotype** | **EUD** | **EUW** | **ASD** | **ASW** |
| --- | --- | --- | --- | --- |
| Hap1 | 0.42 | 0 | 0.081 | 0 |
| Hap2 | 0.356 | 0.977 | 0.096 | 0 |
| Hap3 | 0.099 | 0.023 | 0.281 | 0.174 |
| Hap4 | 0.017 | 0 | 0.162 | 0.13 |
| Hap5 | 0.015 | 0 | 0.099 | 0.109 |
| Hap6 | 0.004 | 0 | 0.068 | 0.109 |
| Hap7 | 0.018 | 0 | 0.004 | 0.022 |
| Hap8 | 0.007 | 0 | 0.035 | 0 |
| Hap9 | 0 | 0 | 0.05 | 0.196 |
| Hap10 | 0 | 0 | 0.039 | 0.043 |
|  | 0.936 | 1 | 0.915 | 0.783 |
