# Supplementary material for: Functional and evolutionary analysis of host Synaptogyrin-2 in porcine circovirus type 2 susceptibility
Source: PLoS Genet. 2023 Nov 27;19(11):e1011029. doi: 10.1371/journal.pgen.1011029 (PMC10703400; doi:10.1371/journal.pgen.1011029)
Supplement: Attachment — Submitted filename: Cover_letter_10102023_final.pdf [file pgen.1011029.s003.pdf]

Editorial Board
PLoS Genetics

Dear Dr. Groenen,

Thank you for the review and comments of our manuscript. We were pleased to see that in general all the reviewers complimented our genetic study stating that, "*this is an interesting study*" (Reviewer 1 and 2), "*it nicely uses a variety of developed genetic tools*", "*well executed, logical, and appropriately interpreted*", "*clearly written and easy to follow*", "*of interest to the broad audience of Plos Genetics*" (Reviewer 1), "*I have really enjoyed reviewing this original research manuscript*"(Reviewer 3), etc. In this version of the manuscript, we addressed the comments and suggestions of the reviewers. Specifically, we made changes to the title to reflect the main focus and results of the study (Reviewer 2), provided additional details of the cell culture experiments, (Reviewer 2), establishment and validation of the cell edited models (Reviewer 3). We discussed in more detail the introgression of European wild boar into domestic herds as a potential source of limited genetic variation at the *SYNGR2* locus in Iberian pigs (Reviewer 3).

Taking in account the recent broad interest of the role of host genetics in viral infections, our study provides not only direct evidence of the role of a functional polymorphism (*SYNGR2 p.Arg63Cys*) in viral disease susceptibility, but also a novel component of the host-virus evolutionary arms race across mammals with *SYNGR2 p.Arg63Cys* representing a species-specific example of putative adaptive evolution. Given the broad appeal of these discoveries and changes implemented following peer review, we hope you will find our paper improved and suitable for PloS Genetics. In the following section we describe in detail the comments of the reviewers and how we addressed them in the updated version of our manuscript.

Sincerely,

Daniel C. Ciobanu, PhD
Professor of Molecular Genetics
University of Nebraska, Lincoln.

REVIEWER 1.
<u>No comments.</u>

REVIEWER 2.
<u>Comment 1.</u> "the tag of the publication "A Model of Host Adaptive Evolution to Viral Pathogens" is a bit lurid and misleading and with the comparisons across species not core to the study."

<u>Response.</u> We agree with Reviewer 2's comment regarding the manuscript title and as such suggest a new title to better fit the focus of the study: "Functional and Evolutionary Analysis of Host Synaptogyrin-2 in Porcine Circovirus Type 2 Susceptibility".

<u>Comment 2.</u> "Figure 1: "three independent replicates": does this mean replicates of qPCR reactions; 12-well plates were used for cell culture, so 12 replicates per clone (supernatant, cell pellet) can be expected; I assume it is 12 wells divided by 4 times points; this needs to be clearly shown".

<u>Response.</u> We thank Reviewer 2 for the comment and pointing out this ambiguity. The "three independent replicates" refers to the number of infection replicates and not qPCR replicates. We have updated the figure legends to "three independent infection replicates" to help clarify this matter. We also added the following sentence in the corresponding materials and methods section: "Infections were conducted three times, each at a different time and cell passage, to obtain independent infection replicates". The use of technical triplicates for qPCR analysis has already been described in the corresponding material and methods section.

REVIEWER 3.
<u>Comment 1.</u> "I missed the validation of knock-out and of the editing. I therefore request that PCR validation, Western-blot, and Sanger sequencing validation would be added to the manuscript. These should be included even if as supplemental material since these are the basis for the whole manuscript."

<u>Response.</u> Thank you for the comment. PCR validation of the deletion in the predicted *SYNGR2* knock-out PK15 clone was provided in our previous publication with a gel image comparing the size of the *SYNGR2* transcript in the wildtype and edited knock-out PKl5 cell lines (Figure 10; Walker et al. 2018). We also provided evidence of nonsense-mediated mRNA decay in the predicted knock-out cell line quantified via qPCR (Figure S9; Walker et al. 2018). To address the Reviewer's concerns, we added a supplementary figure to this paper (Figure S2) depicting the alignment of the *SYNGR2* coding sequence obtained via Sanger sequencing for the wildtype ($wtSYNGR2^{+p.63Arg}$) and edited ($emSYNGR2^{+p.63Cys}$, $2emSYNGR2^{+p.63Cys}$, and $emSYNGR2^{-del}$) PK15 cell lines utilized in this study.

<u>Comment 2.</u> "Discussion does not mention the fact that there is continuous genetic introgression of EWB into Iberian pigs, and recent studies have shown how some samples in ENA cluster closer with EWB and not with other ED. I believe that is more a cause for this result than the fact that it is not introgressed with Asian."

Response. We agree with the reviewer that the high frequency of the ancestral haplotype (Hap2) in Iberian is likely due to the continuous introgression with European Wild Boar (EWB), which were nearly fixed for *Hap2*. We have updated this part of the discussion as follows: "Additional insight is provided by the presence of only *Hap1* and *Hap2* in Iberian samples. Coinciding with noted continuous introgression between Iberian and European wild boar [12, 18-20], *Hap2* was present at the highest frequency in Iberian (88.2%) compared to all other European breeds. However, Iberian pigs are also one of the few European breeds with no known written or molecular evidence of Asian introgression [11, 12]. Therefore, we propose that the favorable *SYNGR2 p.63Cys* allele arose post-domestication in ancestral European domestic pigs from which modern European breeds were formed (Figure 5)."
